# Effects of acute caffeine ingestion on post-activation performance enhancement following individualized drop-jump conditioning in high-level male basketball players: a randomized, double-blind, placebo-controlled crossover trial

**DOI:** 10.3389/fphys.2026.1950037

**Published:** 2026-09-24

**Authors:** Jinda Li, Mingrui Wang, Jiaxuan Sun, Chunlei Li, Junqi Wu, Adrián Rubio, Lun Chen, Zhengsheng Yang, Pengzhou Chen, Yuting Zhou, Mingze Li, Mingjia Li

**Affiliations:** 1 School of Strength and Conditioning Training, Beijing Sport University, Beijing, China; 2 Department of Exercise Physiology, Beijing Sport University, Beijing, China; 3 Liaoning Shenyang Sansheng Flying Leopards Basketball Club, Shenyang, Liaoning, China; 4 Top-Tier Performance Physical Fitness Training Center, Beijing, China; 5 China Basketball College, International Joint Laboratory on High Performance Sports Research, Beijing Sport University, Beijing, China; 6 School of Sports Training, Tianjin University of Sport, Tianjin, China; 7 China Volleyball College, Beijing Sport University, Beijing, China; 8 China Football College, Beijing Sport University, Beijing, China

**Keywords:** athletic performance, muscle strength, physical conditioning, plyometric exercise, sports nutritional physiological phenomena, team sports

## Abstract

**Background:**

Post-activation performance enhancement (PAPE) is widely used as a pre-performance strategy to improve explosive performance. However, whether acute caffeine ingestion can further enhance PAPE following an individualized conditioning stimulus based on athletes’ reactive strength profiles remains unclear. This study investigated the effects of acute caffeine ingestion on PAPE responses induced by a reactive strength index (RSI)-guided individualized drop-jump (DJ) protocol in high-level basketball players.

**Methods:**

Twenty-one male National Athlete Grade I basketball players (Tier 3: Highly Trained/National Level) completed a randomized, double-blind, placebo-controlled crossover trial. Participants performed two experimental conditions involving caffeine ingestion (3 mg·kg^-1^ body mass) or placebo, combined with an RSI-guided individualized DJ conditioning protocol. Countermovement jump (CMJ) performance was assessed before and after conditioning at 15 s, 3 min, 6 min, 9 min, and 12 min. PAPE was defined as the change in CMJ height relative to the post-warm-up baseline.

**Results:**

Individualized DJ conditioning induced a significant time-dependent change in CMJ performance, demonstrating a transient PAPE response (time effect, p < 0.001). However, caffeine ingestion did not significantly modify the temporal profile of PAPE compared with placebo (condition × time interaction, p > 0.05). No significant differences were observed between conditions in peak CMJ height, peak enhancement magnitude, time to peak performance, or cumulative PAPE responses (all p > 0.05). Individual response analysis indicated substantial variability in caffeine-related changes, while sensitivity analysis confirmed the stability of the temporal response pattern.

**Conclusion:**

Acute caffeine ingestion at 3 mg·kg^-1^ body mass did not provide additional ergogenic benefits beyond RSI-guided individualized DJ conditioning in high-level male basketball players. These findings suggest that individualized conditioning strategies may already optimize PAPE responses, with limited additional enhancement from acute caffeine supplementation under the investigated conditions.

## 1 Introduction

Basketball is an intermittent, high-intensity sport characterized by rapid transitions between offense and defense and frequent explosive movements, including sprinting, jumping, landing, cutting, and deceleration (Petway et al., 2020; Taylor et al., 2017). Previous studies have shown that elite professional basketball players perform approximately 40–60 jumps during a single game. Among playing positions, forwards and centers complete an average of approximately 55 jumps per game, whereas guards perform around 45. Jumping actions occur throughout the course of competition and are directly involved in critical game situations, including rebounding, shot blocking, shot finishing, and second-chance scoring opportunities (Ben Abdelkrim et al., 2007; Scanlan et al., 2011; Stojanović et al., 2018; Talpey et al., 2021). Particularly during closely contested games or the closing stages of competition, even small differences in jump height may determine whether a shot successfully avoids a defensive block, whether a rebound is secured, or whether a critical offensive possession is converted into game-decisive points. Among the available assessment tools, the countermovement jump (CMJ) is widely recognized as a valid and reliable measure of lower-limb vertical jumping ability and neuromuscular performance in basketball players and has been extensively used for athletic performance evaluation and training monitoring (Claudino et al., 2017; Heishman et al., 2020; Morrison et al., 2022). Therefore, practical strategies capable of acutely enhancing neuromuscular readiness before competition while aligning peak jumping performance with the temporal demands of competition may provide substantial performance benefits for high-level basketball players. One such approach is post-activation performance enhancement (PAPE), which provides a framework for acutely optimizing explosive performance after a strategically selected conditioning activity.

Post-activation performance enhancement refers to a transient improvement in explosive athletic performance that occurs following a conditioning activity and has been widely investigated as a pre-performance strategy. This phenomenon may enhance short-term performance in jumping, sprinting, throwing, and other ballistic movements through mechanisms involving increased muscle temperature, enhanced neural activation, changes in muscle water content, and the interaction between fatigue- and potentiation-related processes (Alves et al., 2021; Blazevich and Babault, 2019; Seitz and Haff, 2016; Xu et al., 2025). In basketball players, various conditioning activities (CAs) have been shown to elicit PAPE responses, including loaded jump squats, lateral shuffle drills, and high-load upper-body resistance exercises (Dello Iacono et al., 2020; Eken et al., 2022; Gepfert et al., 2020). However, these approaches may also induce substantial residual fatigue and often require specialized training equipment that may not be readily available in pre-competition settings (Maloney et al., 2014; Seitz and Haff, 2016; Xu et al., 2025). Thus, beyond establishing that a conditioning activity can elicit PAPE, an important practical consideration is whether the stimulus can be appropriately individualized to the athlete. Although different conditioning activities can be prescribed using various intensity, volume, or movement-specific parameters, the specific characteristics of the stimulus that can be individualized may differ across exercise modalities (Cengizel, 2025; dos Santos Silva et al., 2023; Maloney et al., 2014; Moura Zagatto et al., 2022; Seitz and Haff, 2016). Accordingly, RSI-guided DJ conditioning represents a practically attractive approach for tailoring the conditioning stimulus to individual reactive strength characteristics while preserving the central objective of PAPE: maximizing subsequent explosive performance without imposing excessive fatigue. Importantly, because the magnitude and duration of PAPE may change over the recovery period, examining the response at multiple post-conditioning time points may provide a more comprehensive characterization of the performance response elicited by an individualized DJ stimulus.

Although the selection and individualization of the conditioning stimulus are important determinants of the PAPE response, the magnitude and time course of post-conditioning performance may also be influenced by nutritional factors. Acute caffeine ingestion represents an important nutritional strategy that may modulate performance responses following a conditioning activity (Filip-Stachnik et al., 2022; Guest et al., 2021; Lazić et al., 2022; Salinero et al., 2019). A substantial body of research has demonstrated that caffeine ingestion can enhance various basketball-specific performance outcomes, including sprinting ability, jumping performance, and repeated high-intensity exercise capacity (Lazić et al., 2022; Li et al., 2024; Salinero et al., 2019). Recent evidence suggests that acute caffeine ingestion may enhance performance responses when combined with PAPE-inducing conditioning activities. In male collegiate basketball players, 5 mg·kg^-1^ of caffeine combined with a plyometric PAPE protocol produced greater improvements in Wingate peak power, mean power, and total work than the same protocol with placebo (Ma et al., 2026). In contrast, 6 mg·kg^-1^ of caffeine did not further enhance CMJ performance following a velocity-controlled back-squat conditioning activity in female volleyball players (Filip-Stachnik et al., 2022). These discrepant findings suggest that the effects of caffeine combined with PAPE may depend on the caffeine dose, conditioning stimulus, and athlete characteristics. Thus, caffeine may serve as a potential adjunct to individualized conditioning rather than as an isolated ergogenic intervention.

The temporal dynamics of PAPE warrant particular consideration because post-conditioning performance is not static but evolves across recovery as potentiation and fatigue interact over time. The magnitude and timing of PAPE may be influenced by recovery duration, conditioning-activity characteristics, and athlete training status (Blazevich and Babault, 2019; Xu et al., 2025). This dynamic perspective is consistent with the broader framework of Network Physiology of Exercise, which conceptualizes acute exercise responses as dynamic interactions among physiological systems operating across multiple temporal scales rather than as isolated static measurements (Balagué et al., 2020, 2022). Accordingly, a single post-conditioning time point may capture only part of an evolving performance trajectory and may fail to identify transient changes in the magnitude or timing of PAPE. Characterizing performance across multiple recovery intervals may therefore provide a more comprehensive representation of the temporal profile of PAPE following an acute conditioning stimulus.

However, previous studies have largely examined caffeine ingestion or DJ conditioning separately, and the effects of their combination on the temporal and multidimensional characteristics of PAPE remain unclear. Specifically, it is unknown whether acute caffeine ingestion can provide additional benefits when DJ height is individualized according to an athlete’s reactive strength capacity. This question is particularly relevant in high-level basketball players, whose extensive training experience, well-developed neuromuscular capacity, and distinct potentiation–fatigue balance may influence the magnitude, timing, and trajectory of post-conditioning performance responses (Stojanovic et al., 2012; Wilson et al., 2013). Although individualized DJ height may optimize the conditioning stimulus, the resulting PAPE response may still vary in magnitude and timing across individuals and recovery periods. Characterizing these temporal and inter-individual responses may therefore clarify whether caffeine provides additional benefits beyond individualized conditioning.

A caffeine dose of 3 mg·kg^-1^ body mass was selected because it represents the lower boundary of the commonly recommended ergogenic range and has been shown to influence exercise and neuromuscular performance, while potentially reducing the adverse effects associated with higher doses (Grgic et al., 2020; Guest et al., 2021; Pickering and Kiely, 2019a). Accordingly, the present study aimed to determine whether acute caffeine ingestion provides an additional ergogenic effect on the multidimensional response characteristics of CMJ performance following RSI-guided individualized DJ conditioning in high-level male basketball players. Specifically, we examined the temporal response profile, peak performance magnitude, time to peak performance, cumulative response, and inter-individual variability in PAPE responses. We hypothesized that, compared with placebo, acute caffeine ingestion would modify the temporal trajectory of CMJ performance following individualized DJ conditioning and confer an additional enhancement in performance beyond individualized conditioning alone, reflected by greater peak CMJ performance and PAPE magnitude during the post-conditioning recovery period. We also anticipated inter-individual variability in the magnitude and timing of the PAPE response.

## 2 Materials and methods

### 2.1 Participants

All participants were certified National Athlete Grade I basketball players in China with prior experience competing in national-level basketball competitions. According to the athlete classification framework proposed by McKay et al. (2022), participants were classified as Tier 3 athletes (Highly Trained/National Level), reflecting their systematic training background and established competitive experience at the national level.

The inclusion criteria were as follows: (a) aged 18–25 years; (b) ≥8 years of systematic basketball training experience, with a minimum training frequency of four sessions per week and an average duration of approximately 1.5–2 h per session; (c) ability to safely complete the standardized warm-up, individualized conditioning activity (CA), and CMJ assessments under investigator supervision; (d) no history of cardiovascular, metabolic, or musculoskeletal disorders; (e) no contraindications to high-intensity exercise or caffeine ingestion; and (f) willingness and ability to comply with all pre-experimental standardization requirements throughout the study, including caffeine and alcohol abstinence, avoidance of moderate- to high-intensity physical activity, maintenance of a regular sleep schedule, and adherence to the standardized dietary and testing-time requirements. Written informed consent was obtained from all participants before enrollment. The exclusion criteria were as follows: (a) lower-limb musculoskeletal injury or surgery within the previous 6 months; (b) chronic pain or neuromuscular disorders; (c) use of medications, nutritional supplements, or ergogenic aids known to affect neuromuscular function, cardiovascular function, or exercise performance within the preceding 4 weeks; (d) known caffeine hypersensitivity, intolerance, or other contraindications to caffeine ingestion; (e) any medical or physical condition that, in the investigators’ judgment, could compromise participant safety or the integrity of the experimental procedures; and (f) failure to comply with the predefined pre-experimental standardization requirements before any experimental session, including the prescribed caffeine and alcohol abstinence, exercise restriction, minimum sleep duration, dietary restrictions, or scheduled testing time. Compliance was assessed using a standardized checklist before each experimental session, and data from sessions that did not meet the predefined compliance requirements were excluded from the final analysis.

The eligibility criteria were established to ensure participant safety, minimize potential confounding factors, and maintain standardized experimental conditions while accommodating normal variations in training cycles and competitive schedules. All participants reported training at least four times per week, consistent with the demands of high-level basketball. Participant characteristics are presented in **Table 1**.

**Table 1:** Characteristics of participants.

Sample size	Athletic level	Age (years)	Height (cm)	Weight (kg)	Training experience (years)
21	Tier 3 (Highly Trained/National Level)	21.91 ± 1.45	193.35 ± 5.48	91.73 ± 9.69	10.64 ± 1.63

Values are presented as mean ± SD. Athletic level was classified according to the athlete classification framework proposed by McKay et al., with Tier 3 representing the Highly Trained/National Level category. All participants were male National Athlete Grade I basketball players with prior experience in national-level competition.

The study was conducted in accordance with the Declaration of Helsinki and approved by the Sports Science Experiment Ethics Committee of Beijing Sport University (Approval No. 2026157H). Written informed consent was obtained from all participants before participation, and participants were informed of their right to withdraw from the study at any time without penalty. The trial was registered with the Chinese Clinical Trial Registry (registration number: ChiCTR2600130746).

### 2.2 Experimental design

The study employed a randomized, double-blind, placebo-controlled crossover design, in which each participant completed both the caffeine (CAF) and placebo (PLA) conditions on separate testing days. The order of the experimental conditions was counterbalanced using a randomized allocation sequence, and the two sessions were separated by a 1-week washout period to minimize potential carryover effects (Guest et al., 2021).

To minimize potential order effects, a 2 × 2 Latin square crossover design with counterbalanced treatment order was used. Twenty-one participants were randomly assigned, using a computer-generated randomization sequence created with an online randomization tool, to one of two balanced treatment sequences: Sequence 1 (Week 1: PLA + PAPE; Week 2: CAF + PAPE) or Sequence 2 (Week 1: CAF + PAPE; Week 2: PLA + PAPE) (RANDOM.ORG, n.d). Randomization and allocation concealment were performed by an independent researcher who was not involved in participant recruitment, data collection, or outcome assessment. The allocation schedule was securely sealed and remained concealed until completion of the study (Ma et al., 2026).

Previous studies have used standardized familiarization procedures and jumping instructions for CMJ and DJ testing, while individualized DJ protocols based on the reactive strength index (RSI) have also been employed to identify athlete-specific optimal drop heights (Byrne et al., 2010; Cengizel, 2025; Seitz and Haff, 2016; Young et al., 1995). Participants completed a 2-week familiarization period to standardize CMJ and DJ testing procedures, with particular emphasis on take-off mechanics and optimal force application timing. During the familiarization period, each participant completed an incremental DJ assessment to determine their individualized optimal DJ drop height. Specifically, participants performed three maximal-effort DJs from each of five drop heights (20, 30, 40, 50, and 60 cm) using force plates (Kunwei Sports Technology Corporation, China). The KunWei force measurement system has demonstrated strong concurrent validity and reliability compared with a laboratory-standard Bertec force platform, with intraclass correlation coefficients (ICC) exceeding 0.98 for force-time curve measurements and normalized root mean square error values below 0.165 (Hao et al., 2024). Throughout testing, participants were instructed to keep their hands on their hips and to jump as high as possible while minimizing ground contact time. RSI was calculated separately for each DJ trial as jump height divided by ground contact time. Consistent with the RSI-based method described by Byrne et al. (2010) and Cengizel (2025), the individualized optimal DJ drop height was defined as the drop height associated with the highest single-trial RSI observed across all five tested heights. Accordingly, the optimal height was selected on the basis of the maximum individual RSI value rather than the mean RSI of the three trials performed at each height. All technically valid trials were retained for determination of the optimal height, and trial-to-trial variability alone was not used as an exclusion criterion. No ties in the maximum RSI value occurred across the tested heights; therefore, no secondary tie-breaking criterion was required.

Individualized caffeine doses were determined from each participant’s body mass measured during the DXA assessment using a dual-energy X-ray absorptiometry (DXA) system (XR-46, Norland, USA). In the CAF condition, participants received 3 mg·kg^-1^ body mass of anhydrous caffeine (Nutricost, USA), a dose selected at the lower boundary of the commonly recommended ergogenic range and previously shown to influence neuromuscular and jumping performance (Guest et al., 2021; Stojanovic et al., 2012). All caffeine supplements used throughout the study were obtained from the same product batch to ensure consistency in caffeine source and preparation. Supplement preparation was conducted by an independent researcher who had no involvement in participant testing, experimental data collection, or statistical analyses. For each participant, the required caffeine dose was calculated individually based on body mass, after which the corresponding amount of caffeine was carefully weighed using a precision electronic balance (Sanliang AB303, Japan; readability: 0.001 g [1 mg]). The weighed caffeine was then transferred into standardized empty gelatin capsules. In the PLA condition, food-grade starch was prepared using the same procedure and placed into identical capsules. The CAF and PLA capsules were matched in appearance, size, packaging, labeling, and administration procedures to maintain blinding throughout the experiment. The same caffeine batch and standardized preparation procedures were used across all experimental sessions to minimize potential variability in supplement preparation and ensure consistent individualized dosing (Salinero et al., 2019; Zhu et al., 2025). Following generation of the randomization sequence, supplements were coded according to the predetermined treatment allocation, with the allocation code retained by the independent researcher responsible for supplement preparation. Participants and testing personnel remained blinded to treatment assignment throughout data collection, and the code was disclosed only after completion of all experimental sessions and prespecified primary data processing. Supplement ingestion was directly supervised, and complete consumption was confirmed before the standardized 60-min period preceding performance testing, resulting in 100% ingestion compliance across all initiated sessions. Participants were monitored for supplementation-related symptoms or discomfort. Three non-serious subjective events were reported: one participant experienced a subjective sensation of accelerated heartbeat during the PLA condition, although concurrent Polar heart-rate monitoring showed no apparent abnormal elevation; after reassessment, the participant safely completed the session. Two participants reported delayed sleep onset at approximately 05:00–06:00 following the CAF condition. None of these events required medical intervention, session termination, or participant withdrawal, and no serious adverse events occurred.

The interval from capsule ingestion to completion of the conditioning activity (CA) was standardized to approximately 60 min. Following the standardized pre-conditioning procedures, participants performed the CA, which consisted of the RSI-guided individualized drop-jump (DJ) protocol. The CA comprised three sets of five maximal-effort DJs, with a 1-min passive recovery interval between sets, resulting in a standardized total volume of 15 DJs for all participants. The three DJ sets were considered a single conditioning stimulus rather than separate interventions. Accordingly, the CA was completed approximately 60 min after capsule ingestion. Following completion of the final DJ repetition, the first post-conditioning CMJ assessment was performed 15 s later, corresponding to approximately 60.25 min after capsule ingestion. Subsequent CMJ assessments were performed at 3, 6, 9, and 12 min after completion of the final DJ repetition, corresponding to approximately 63, 66, 69, and 72 min after capsule ingestion, respectively. Thus, the ingestion-to-CA-completion interval was approximately 60 min, whereas the ingestion-to-first-post-conditioning-CMJ interval was approximately 60.25 min. The timing of the CA and all subsequent CMJ assessments was standardized across the caffeine and placebo conditions. The CA structure was selected to provide a sufficient neuromuscular stimulus while limiting excessive fatigue accumulation, consistent with previous PAPE investigations using ballistic conditioning activities (Cengizel, 2025; dos Santos Silva et al., 2023; Xu et al., 2025).

Each experimental session followed an identical standardized protocol consisting of pre-experimental status assessment, a standardized warm-up, the conditioning activity, and subsequent CMJ assessments. CMJ performance was evaluated before the warm-up, immediately after the warm-up, and at 15 s, 3 min, 6 min, 9 min, and 12 min after completion of the CA. Two CMJ trials were performed at each time point, and the highest jump was retained for statistical analysis (**Figure 1**).

**Figure 1:**
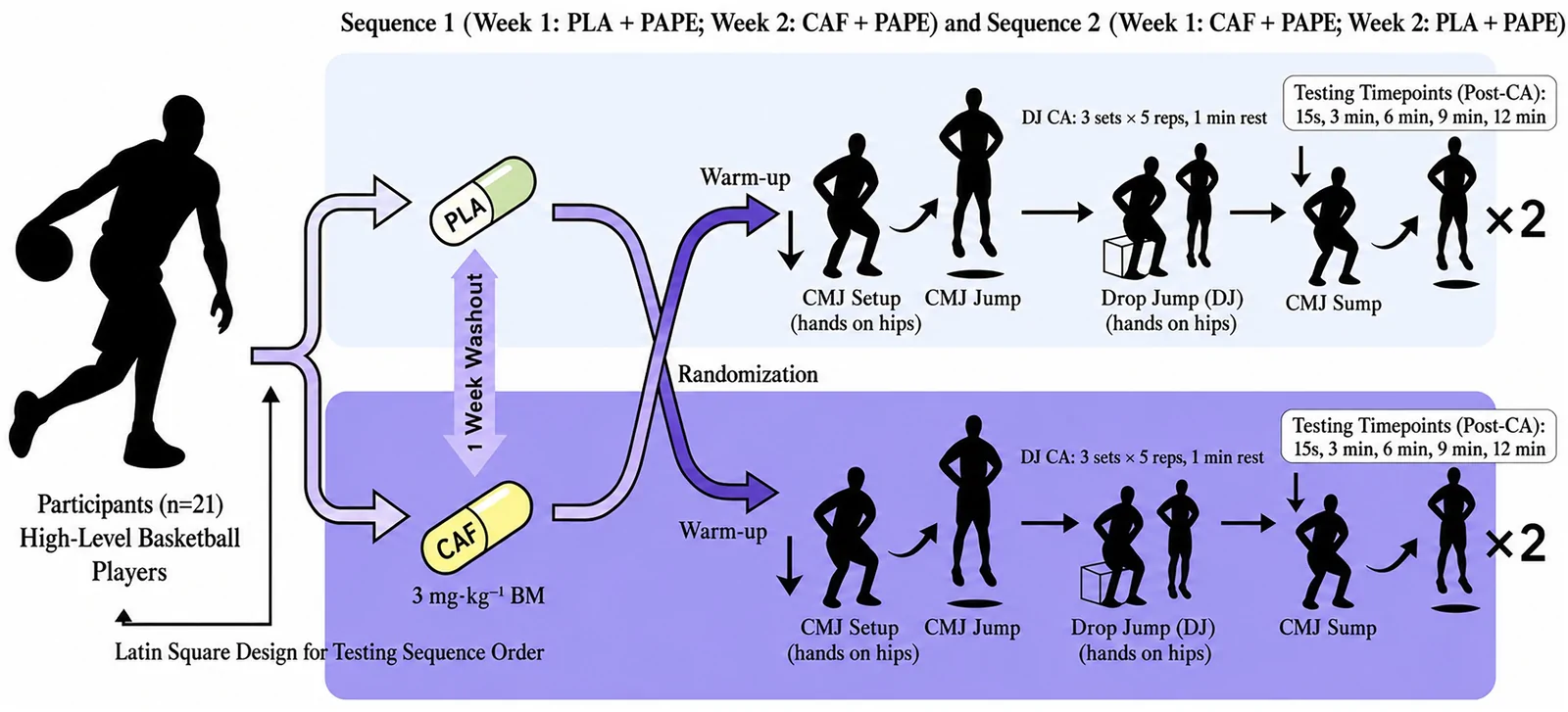
Experimental design and study procedures. Schematic illustration of the randomized, double-blind, placebo-controlled crossover design. Following a 2-week familiarization period, participants completed two experimental trials (caffeine and placebo conditions) separated by a 1-week washout period. Approximately 60 min after capsule ingestion, participants performed a standardized warm-up followed by an RSI-guided individualized drop-jump (DJ) conditioning activity (three sets of five DJs). Countermovement jump (CMJ) performance was assessed before warm-up, immediately after warm-up, and at 15 s, 3 min, 6 min, 9 min, and 12 min following completion of the conditioning activity.

### 2.3 Warm-up and PAPE potentiation protocol

#### 2.3.1 Standardized dynamic warm-up

Following the protocols described by dos Santos Silva et al. (2023) and Cengizel (2025), all participants completed a standardized dynamic warm-up consisting of two consecutive phases designed to optimize neuromuscular readiness before performance testing.

The first phase comprised 5 min of low-intensity jogging, during which heart rate was maintained at approximately 120 beats·min^-1^ and continuously monitored using a Polar V800 training computer paired with a Polar H7 heart rate sensor (Polar Electro Oy, Kempele, Finland) to standardize internal exercise intensity. The second phase consisted of approximately 5 min of standardized dynamic mobility exercises targeting the major joints of the lower extremities (ankle, knee, and hip) and their associated musculature. These exercises included knee-to-chest raises with plantar flexion, figure-four squats, ankle circles, and the “world’s greatest stretch.” The duration and sequence of each exercise were standardized across participants, resulting in an approximate total warm-up duration of 10 min. This standardized warm-up protocol was used to establish a consistent physiological and neuromuscular state before the conditioning activity and subsequent CMJ assessments (Cengizel, 2025; dos Santos Silva et al., 2023).

#### 2.3.2 Individualized PAPE conditioning protocol

The DJ was performed from an individually determined optimal drop height, established during the familiarization period using the RSI as part of the individualized PAPE protocol. Participants stood on the box with their feet shoulder-width apart and their hands positioned on their hips throughout the movement to eliminate the contribution of arm swing. They were instructed to step forward from the box without jumping upward, land simultaneously on both feet, and immediately execute a maximal vertical rebound jump upon ground contact. During landing, participants maintained a stiff yet controlled lower-limb posture while minimizing excessive knee and hip flexion to facilitate a rapid transition from the eccentric to the concentric phase and maximize utilization of the stretch–shortening cycle (SSC). Any trial involving loss of balance, excessive countermovement, or arm swing was discarded and repeated. The conditioning activity consisted of three sets of five DJs, with 1 min of passive recovery between sets to standardize recovery throughout the protocol (dos Santos Silva et al., 2023; Maloney et al., 2014).

### 2.4 Countermovement jump testing procedure

CMJ performance was assessed as an indicator of lower-limb explosive performance at multiple time points following the CA. A standardized data collection procedure was implemented using the same force plates described above. Consistent with established methodological recommendations for CMJ assessment and PAPE research, participants performed all CMJ trials with their hands placed on their hips throughout the movement to minimize the contribution of arm swing and improve measurement consistency. Each trial began from an upright standing position, followed by a rapid countermovement to a self-selected depth and an immediate maximal vertical jump. Participants were instructed to maximize jump height while maintaining consistent movement execution across all testing sessions (dos Santos Silva et al., 2023; Maloney et al., 2014; Seitz and Haff, 2016).

CMJ assessments were performed before the warm-up, immediately after the standardized warm-up, and at 15 s, 3 min, 6 min, 9 min, and 12 min following completion of the CA. Two CMJ trials were completed at each time point, with a standardized 30-s passive recovery interval between trials. This recovery interval was kept consistent across all participants, assessment time points, and experimental sessions to minimize the influence of acute fatigue while maintaining a standardized testing procedure. The highest jump achieved at each time point was retained for subsequent statistical analysis, consistent with common CMJ assessment procedures aimed at capturing maximal neuromuscular performance while reducing the influence of occasional submaximal attempts and trial variability (Duan et al., 2024; Heishman et al., 2020).

This repeated-measures protocol was designed to characterize the temporal trajectory of PAPE responses across multiple recovery intervals. By assessing CMJ performance at several post-conditioning time points, this approach allowed the identification of dynamic changes in performance enhancement and fatigue-related responses over time rather than relying on isolated single-time-point measurements (Balagué et al., 2020, 2022).

### 2.5 Pre-experimental standardization, compliance monitoring, and blinding assessment

To minimize potential confounding factors and ensure comparable physiological and metabolic conditions across testing sessions, a standardized pre-test control protocol was implemented. Throughout the study, participants were instructed to maintain their habitual dietary intake and training routines.

During the 24 h preceding each experimental session, participants were required to abstain from all caffeine-containing products, including coffee, tea, energy drinks, caffeinated soft drinks, chocolate, and caffeine-containing nutritional supplements, as well as from alcohol consumption (Guest et al., 2021).

To minimize the influence of residual fatigue on neuromuscular performance, participants refrained from moderate- to high-intensity physical activity for at least 48 h before each testing session. They were also instructed to maintain their habitual sleep schedule and obtain a minimum of 7 h of sleep on the night before testing. To reduce potential circadian influences, all experimental sessions for each participant were conducted at the same time of day (± 1 h).

Participants consumed their final meal at least 2 h before testing and refrained from any food intake during the final 1 h before arrival at the laboratory. Normal hydration was permitted throughout the standardization period.

Following the conditioning activity, participants remained seated quietly during the intervals between scheduled CMJ assessments. At each prespecified time point (15 s, 3 min, 6 min, 9 min, and 12 min after the conditioning activity), participants performed the CMJ assessments before returning to a seated position until the next assessment. Throughout these intervals, participants were instructed to refrain from stretching, self-massage, and any additional physical activity, and to consume no food or beverages other than water. These procedures were standardized across experimental conditions to minimize potential external influences on post-conditioning responses (Blazevich and Babault, 2019).

Compliance with all pre-test instructions was verified using a standardized checklist completed before each experimental session. Only data from participants who fully complied with the pre-test requirements were included in the final analyses.

To assess the effectiveness of the blinding procedure, immediately after each experimental session, participants were asked to indicate which intervention they believed they had received by selecting one of three response options: “caffeine,” “placebo,” or “unsure” (Boutron et al., 2006; Saunders et al., 2017). Participants’ responses were recorded separately for the PAPE + CAF and PAPE + PLA conditions and compared with the intervention actually administered. Blinding effectiveness was quantified using the Bang Blinding Index (BI), calculated as BI = 2P_correct_ − 1, where P_correct_ represents the proportion of participants who correctly identified the intervention they had received. The BI ranges from −1 to +1, with values below 0 indicating a tendency toward incorrect treatment identification, 0 indicating identification at chance level, and values above 0 indicating a tendency toward correct treatment identification. The BI was calculated separately for the PAPE + CAF and PAPE + PLA conditions.

### 2.6 Data analysis

An *a priori* sample size calculation was performed using G*Power software (version 3.1.9.7; Heinrich Heine University Düsseldorf, Germany) based on the hypothesized condition × time interaction in the repeated-measures ANOVA. The calculation assumed a medium effect size (f = 0.25), an α level of 0.05, statistical power (1 − β) of 0.80, two experimental conditions, six post-conditioning repeated measurements, a correlation of 0.50 among repeated measures, and a nonsphericity correction (ϵ) of 1.0. Under these prespecified assumptions, a minimum sample size of 20 participants was required. Accordingly, 21 participants who completed all experimental conditions were included in the final analysis. These parameters were specified *a priori* before data collection and were not derived from or adjusted according to the observed effect size or sphericity characteristics of the present dataset.

All statistical analyses were performed using SPSS Statistics version 26.0 (IBM, USA). Continuous variables are presented as mean ± standard deviation (Mean ± SD). All statistical tests were two-tailed, and a *P*-value < 0.05 was considered indicative of statistical significance. The primary estimand was defined as the between-condition difference in CMJ performance following individualized DJ conditioning, comparing caffeine and placebo conditions across the post-conditioning period. Secondary estimands included differences in the temporal PAPE response profile, baseline-normalized CMJ changes (ΔCMJ), peak response, and cumulative response quantified by area under the curve (AUC).

The normality of continuous variables was assessed using the Shapiro–Wilk test, and homogeneity of variance was assessed using Levene’s test, where appropriate. Mauchly’s test was used to assess sphericity, with Greenhouse–Geisser corrections applied when the assumption was violated. Absolute CMJ height was analyzed using a two-way repeated-measures ANOVA, with condition (caffeine [CAF] and placebo [PLA]) and time (before the warm-up, immediately after the standardized warm-up, and 15 s, 3 min, 6 min, 9 min, and 12 min after completion of the conditioning activity) as within-subject factors. Baseline-normalized changes in CMJ performance (ΔCMJ) were analyzed separately using a two-way repeated-measures ANOVA, with condition (CAF and PLA) and time (15 s, 3 min, 6 min, 9 min, and 12 min after completion of the conditioning activity) as within-subject factors; ΔCMJ was calculated relative to the post-warm-up baseline. Significant main effects or condition × time interactions were followed by Bonferroni-adjusted pairwise comparisons, where appropriate. Partial eta-squared (ηp²) was reported as the ANOVA effect size. Peak CMJ height, peak CMJ gain, and time-to-peak were determined from the maximal CMJ response across the five post-conditioning nominal assessment time points (15 s, 3 min, 6 min, 9 min, and 12 min). Peak CMJ gain was calculated relative to the post-warm-up baseline. Because two CMJ trials were performed at each nominal assessment time point and the higher value was retained, time-to-peak was defined as the nominal assessment time point at which the highest observed CMJ value occurred. The coefficient of variation (CV%) was calculated from the two CMJ trials at each assessment time point to quantify within-session measurement variability. Peak-response variables were compared between CAF and PLA using paired-samples t-tests, with mean differences and 95% confidence intervals (95% CI) reported (Armstrong, 2014; Maxwell et al., 2017).

To further characterize temporal changes within each condition, paired-samples t-tests with Bonferroni adjustment were performed to compare each post-conditioning time point with the post-warm-up baseline. These peak-response variables were compared between caffeine and placebo conditions using paired-samples t-tests, with mean differences and corresponding 95% confidence intervals (95% CI) reported (Armstrong, 2014; Field, 2005).

To quantify the cumulative PAPE response throughout the recovery period, the area under the curve (AUC) was calculated using the trapezoidal rule based on baseline-normalized CMJ changes. Total, positive, and negative AUC were calculated to represent overall, enhanced, and reduced performance responses, respectively. Because total AUC is derived from the sum of positive and negative AUC components, these variables were considered related outcomes and compared between conditions using paired-samples t-tests.

The temporal pattern of CMJ responses was additionally examined using a linear mixed-effects model (LMM) as a complementary sensitivity analysis to the primary repeated-measures ANOVA. Condition, time, and the condition × time interaction were specified as fixed effects, with participant included as a random intercept. An autoregressive first-order [AR (Petway et al., 2020)] covariance structure was used to account for serial correlation among repeated observations, and an unstructured (UN) covariance structure was examined as a sensitivity analysis. The condition × time interaction was evaluated using likelihood ratio tests, with fixed-effect estimates obtained from the full model using restricted maximum likelihood. LOPO sensitivity analysis was subsequently performed within the same LMM framework to assess whether the temporal findings were disproportionately influenced by any individual participant (Liu et al., 2012).

Exact p-values, effect sizes, and 95% confidence intervals were reported whenever appropriate to facilitate interpretation of both the statistical significance and practical magnitude of the observed effects.

## 3 Results

The effectiveness of the blinding procedure was assessed using the Bang Blinding Index (BI). Five participants correctly identified the placebo intervention in the PAPE + PLA condition, whereas seven participants correctly identified caffeine ingestion in the PAPE + CAF condition. The corresponding BI values were −0.52 and −0.33, respectively. Both values were below zero, indicating a tendency toward incorrect rather than correct treatment identification and suggesting that participants were unable to reliably distinguish between the caffeine and placebo conditions.

### 3.1 Time-course characteristics of CMJ performance following individualized drop-jump conditioning

A two-way repeated-measures ANOVA revealed no significant main effect of condition on absolute CMJ height (F(1,20) = 3.35, p = 0.082, ηp² = 0.144). In contrast, a significant main effect of time was observed (F(6,120) = 30.40, p < 0.001, Greenhouse–Geisser-corrected p < 0.001, ηp² = 0.603), indicating that CMJ performance changed across the post-conditioning recovery period. No significant condition × time interaction was detected (F(6,120) = 0.49, p = 0.814, Greenhouse–Geisser-corrected p = 0.659, ηp² = 0.024).

Similarly, analysis of ΔCMJ relative to the post-warm-up baseline demonstrated a significant main effect of time (F(4,80) = 16.05, p < 0.001, Greenhouse–Geisser-corrected p < 0.001, ηp² = 0.445). However, neither the main effect of condition (F(1,20) = 0.43, p = 0.519, ηp² = 0.021) nor the condition × time interaction (F(4,80) = 0.47, p = 0.759, Greenhouse–Geisser-corrected p = 0.687, ηp² = 0.023) reached statistical significance (**Table 2**).

**Table 2:** Two-way repeated-measures ANOVA results for CMJ performance following individualized DJ conditioning.

Outcome	Source	F (df_1_, df_2_)	p	ηp²
Absolute CMJ height	Condition	3.35 (1, 20)	0.08	0.14
	Time	30.40 (6, 120)	<0.001	0.60
	Condition × Time	0.49 (6, 120)	0.66	0.02
	Condition	0.43 (1, 20)	0.52	0.02
ΔCMJ height	Time	16.05 (4, 80)	<0.001	0.45
	Condition × Time	0.47 (4, 80)	0.69	0.02

The primary estimand was the between-condition difference in CMJ performance following individualized DJ conditioning, comparing caffeine and placebo conditions across the post-conditioning assessment period. Secondary estimands included the effect of caffeine ingestion on the temporal response profile of PAPE (condition × time interaction) and baseline-normalized changes in CMJ performance (ΔCMJ). Two-way repeated-measures analysis of variance (ANOVA) was conducted with condition (caffeine and placebo) and time as within-subject factors. Data are presented as F statistics with numerator and denominator degrees of freedom (df_1_ and df_2_), p-values, and partial eta squared (ηp²). Greenhouse–Geisser corrections were applied when the assumption of sphericity was violated.

### 3.2 Pairwise comparisons of temporal CMJ responses following individualized drop-jump conditioning

Bonferroni-adjusted pairwise comparisons revealed no significant differences in absolute CMJ height between the caffeine and placebo conditions at any post-conditioning time point (all adjusted p > 0.05). CMJ height was numerically higher in the caffeine condition throughout the post-conditioning period, with the largest between-condition difference observed at 6 min (mean difference = 1.52 cm, 95% CI: 0.33–2.72; adjusted p = 0.149), followed by 12 min (mean difference = 1.26 cm, 95% CI: 0.26–2.26; adjusted p = 0.159). Similarly, ΔCMJ did not differ significantly between conditions at any post-conditioning time point (all adjusted p > 0.05), with between-condition differences ranging from 0.05 to 0.71 cm (**Table 3**, **Figure 2**).

**Table 3:** Pairwise comparisons of CMJ performance between caffeine and placebo conditions following individualized drop-jump conditioning.

Outcome	Time point	Caffeine	Placebo	Mean difference (95% CI)	Adjusted p
Absolute CMJ height (cm)	PreWarm	40.03	39.33	0.70 (−0.68, 2.09)	1.000
	PostWarm	43.06	42.25	0.80 (−0.88, 2.49)	1.000
	T15s	44.13	42.79	1.34 (−0.07, 2.75)	0.537
	T3min	44.63	43.78	0.85 (−1.04, 2.74)	1.000
	T6min	43.97	42.45	1.52 (0.33, 2.72)	0.149
	T9min	43.07	41.78	1.30 (−0.08, 2.67)	0.559
	T12min	42.61	41.35	1.26 (0.26, 2.26)	0.159
ΔCMJ height (cm)	T15s	1.07	0.53	0.54 (−0.56, 1.63)	1.000
	T3min	1.58	1.53	0.05 (−1.76, 1.85)	1.000
	T6min	0.91	0.20	0.71 (−0.79, 2.23)	1.000
	T9min	0.01	−0.48	0.49 (−0.99, 1.97)	1.000
	T12min	−0.45	−0.90	0.45 (−0.99, 1.91)	1.000

Data are presented as mean CMJ height (cm). CAF and PLA denote the caffeine and placebo conditions, respectively. Between-condition comparisons at each post-conditioning time point were performed using paired-samples comparisons with Bonferroni correction for multiple testing. Mean differences were calculated as CAF minus PLA and are reported with 95% confidence intervals (CI). Positive values indicate greater CMJ performance in the caffeine condition, whereas negative values indicate greater performance in the placebo condition. ΔCMJ represents the change in CMJ height relative to baseline. PreWarm and PostWarm represent assessments performed before and after the standardized warm-up protocol, respectively; T15s, T3min, T6min, T9min, and T12min indicate assessments conducted at 15 s, 3 min, 6 min, 9 min, and 12 min after the individualized drop-jump conditioning activity. Statistical significance was set at an adjusted p-value < 0.05.

**Figure 2:**
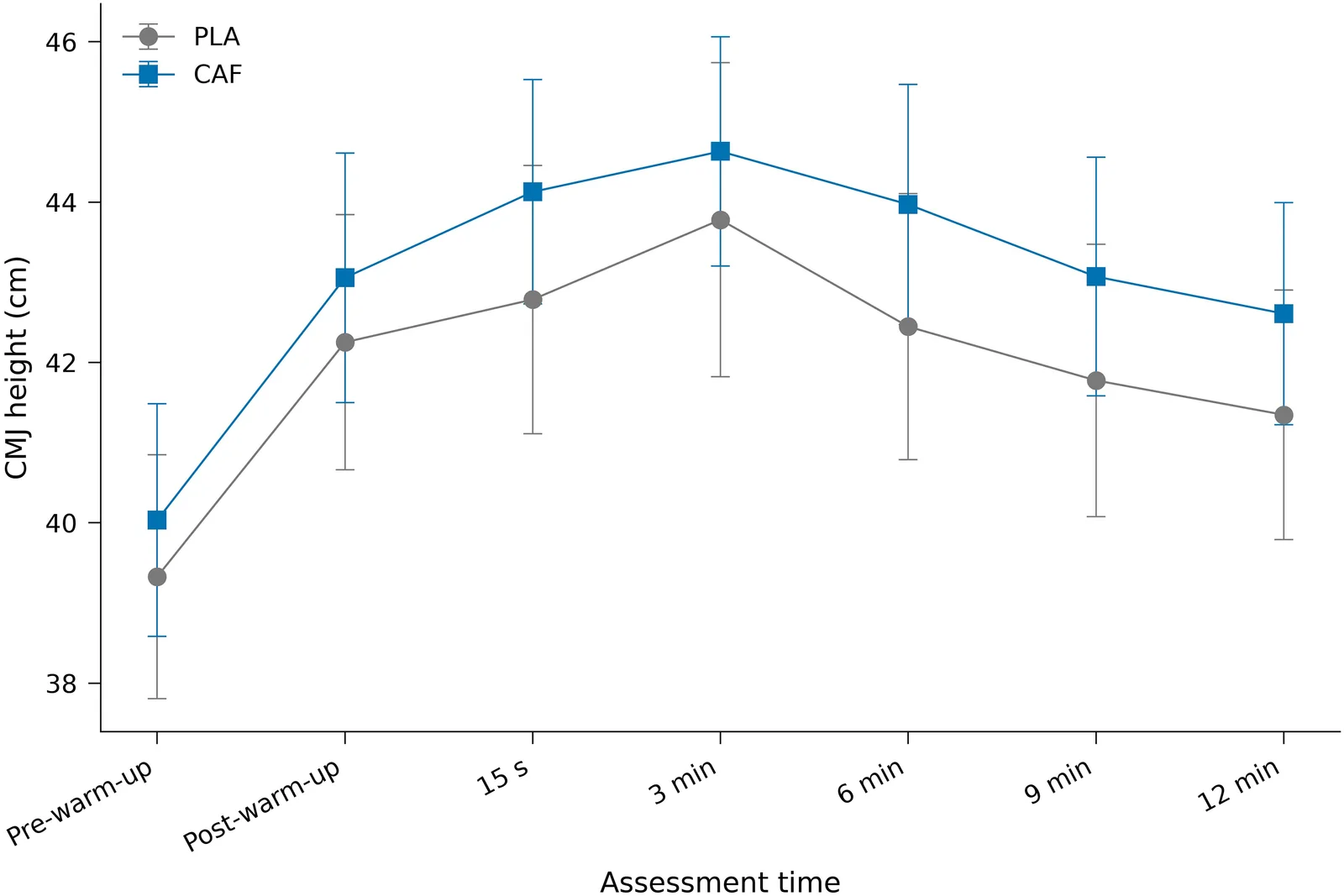
Time course of CMJ performance following individualized DJ conditioning under caffeine and placebo conditions. CMJ assessments were performed before the warm-up, immediately after the standardized warm-up, and at 15 s, 3 min, 6 min, 9 min, and 12 min following completion of the CA. Data are presented as mean ± SEM. Between-condition differences at each time point were evaluated using Bonferroni-adjusted pairwise comparisons following two-way repeated-measures ANOVA. Although CMJ values were consistently numerically higher in the caffeine condition throughout the recovery period, no statistically significant between-condition differences were detected after adjustment for multiple comparisons (all adjusted p > 0.05).

Within-condition comparisons with the post-warm-up baseline showed no significant differences in CMJ height at any post-conditioning time point in either condition after Bonferroni correction (all adjusted p > 0.05). Under caffeine, CMJ height increased numerically at 15 s (mean change = 1.07 cm), 3 min (1.58 cm), and 6 min (0.91 cm), before returning toward baseline at 9 min (0.01 cm) and 12 min (−0.45 cm). A similar temporal pattern was observed under placebo, with numerical increases at 15 s (0.53 cm) and 3 min (1.53 cm), followed by smaller changes at 6 min (0.20 cm), 9 min (−0.48 cm), and 12 min (−0.90 cm). However, none of these within-condition comparisons reached statistical significance after Bonferroni correction (CAF: adjusted p = 0.412–1.000; PLA: adjusted p = 0.227–1.000) (**Figure 3**).

**Figure 3:**
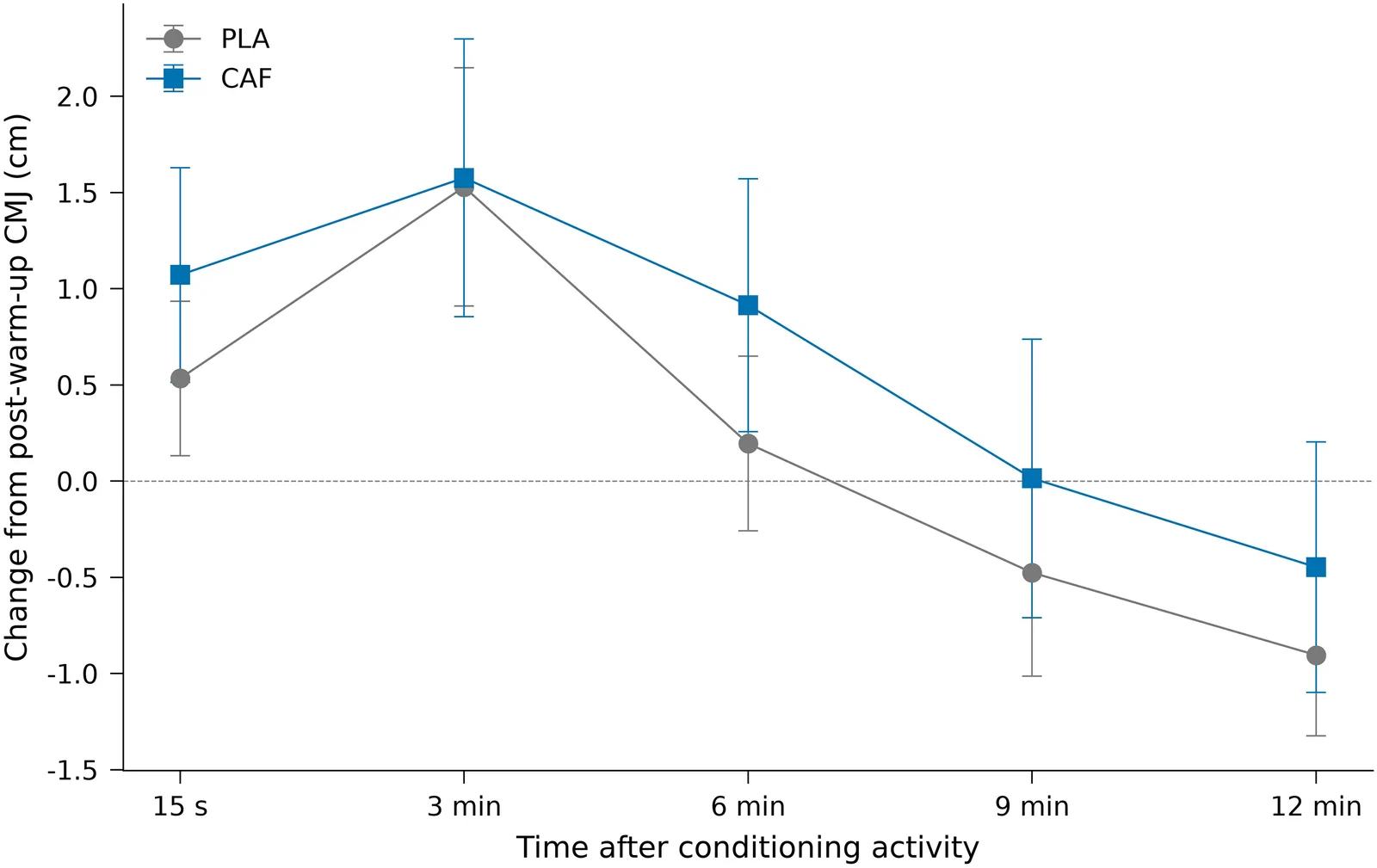
Temporal changes in CMJ performance relative to baseline following individualized DJ conditioning under caffeine and placebo conditions. Changes in CMJ height were calculated relative to the post-warm-up baseline and assessed at 15 s, 3, 6, 9, and 12 min following individualized drop-jump conditioning under caffeine and placebo conditions. Data are presented as mean ± SEM. Within-condition changes from baseline were evaluated using paired-samples t-tests with Bonferroni correction for multiple comparisons. Although both conditions exhibited transient numerical improvements in CMJ performance during the early recovery period, followed by a gradual return toward baseline, no within-condition differences remained statistically significant after adjustment for multiple comparisons (all adjusted p > 0.05).

### 3.3 Peak response characteristics following individualized drop-jump conditioning

Peak response characteristics following individualized drop-jump conditioning are summarized in **Table 4**. The within-session coefficient of variation (CV) between the two CMJ trials ranged from 1.90% to 2.43% across the five post-conditioning time points under the caffeine condition and from 2.25% to 3.36% under the placebo condition, indicating relatively small trial-to-trial variability. No significant between-condition differences were observed for any peak-response variable (all p > 0.05).

**Table 4:** Peak response characteristics of CMJ performance following individualized DJ conditioning under caffeine and placebo conditions.

Outcome	CAF	PLA	Mean difference (95% CI)	t	p
Peak CMJ height (cm)	45.32	44.11	1.21 (−0.62 to 3.05)	1.30	0.209
Peak CMJ gain (cm)	2.27	1.86	0.41 (−1.36 to 2.18)	0.45	0.656
Peak CMJ gain (%)	6.06	4.19	1.87 (−3.09 to 6.83)	0.74	0.469
Time to peak CMJ (min)	2.83	2.51	0.32 (−1.24 to 1.88)	0.40	0.691

Values are presented as mean ± SEM. Mean differences were calculated as caffeine minus placebo and are reported with corresponding 95% confidence intervals (95% CI). Between-condition comparisons were performed using paired-samples t tests. Peak CMJ height, peak CMJ gain, and time to peak CMJ were defined based on each participant’s maximal CMJ response across the five post-conditioning assessment time points (15 s, 3 min, 6 min, 9 min, and 12 min). Peak CMJ gain was calculated relative to the post-warm-up baseline. Statistical significance was set at an adjusted p value < 0.05.

Peak CMJ height was numerically greater following caffeine ingestion than placebo (45.32 vs. 44.11 cm), corresponding to a mean between-condition difference of 1.21 cm (95% CI: −0.62 to 3.05 cm; t(20) = 1.30, p = 0.209). Likewise, peak enhancement relative to the post-warm-up baseline was comparable between conditions, both when expressed as an absolute increase (2.27 vs. 1.86 cm; mean difference = 0.41 cm, 95% CI: −1.36 to 2.18 cm; t(20) = 0.45, p = 0.656) and as a percentage increase (6.06% vs. 4.19%; mean difference = 1.87%, 95% CI: −3.09% to 6.83%; t(20) = 0.74, p = 0.469).

The time required to attain peak CMJ performance was also similar between conditions (2.83 vs. 2.51 min; mean difference = 0.32 min, 95% CI: −1.24 to 1.88 min; t(20) = 0.40, p = 0.691). Descriptively, peak CMJ performance occurred most frequently at 15 s following conditioning under the caffeine condition (47.6%), whereas under placebo the highest proportion of peak responses was observed at 3 min (52.4%). Peak responses at 9 min were observed only following caffeine ingestion (14.3%), whereas no participants reached their peak performance at 9 or 12 min under placebo (**Figure 4**). These descriptive differences in peak timing should not be interpreted as evidence of a significant between-condition effect, given the absence of a significant difference in mean time to peak performance.

**Figure 4:**
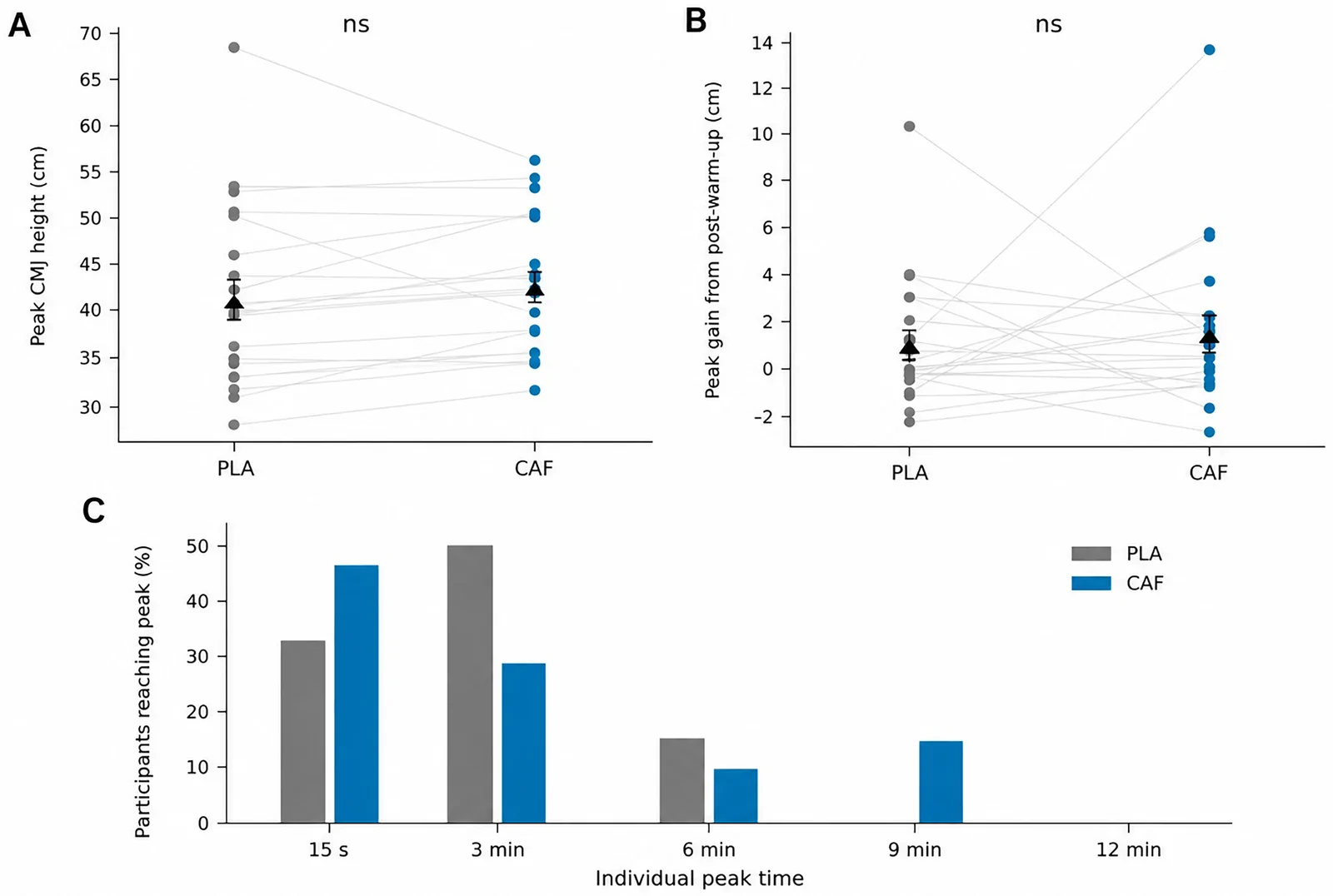
Peak response characteristics of CMJ performance following individualized DJ conditioning under caffeine and placebo conditions. **(A)** Individual peak CMJ height achieved during the 12-min recovery period under PLA and CAF conditions. Peak CMJ height was defined as the highest CMJ value obtained by each participant across the five post-conditioning time points (15 s, 3 min, 6 min, 9 min, and 12 min). Gray connecting lines represent individual participants, whereas black symbols and error bars indicate group mean ± SEM of individual peak responses. **(B)** Individual peak gain in CMJ height relative to the post-warm-up baseline under PLA and CAF conditions. Peak gain was calculated as the maximum change from baseline achieved by each participant across the post-conditioning recovery period. Gray connecting lines represent individual participants, whereas black symbols and error bars indicate group mean ± SEM of individual peak responses. **(C)** Distribution of the time point at which each participant achieved their individual peak CMJ performance during the recovery period. Bars represent the percentage of participants reaching their individual peak response at each post-conditioning time point under PLA and CAF conditions. Peak variables were derived from individual within-subject maxima across the five post-conditioning time points and therefore represent individual maximal responses rather than the maximum value of the group mean temporal curve. No significant between-condition differences were observed for peak CMJ height, peak gain relative to baseline, or the distribution of peak response timing (all p > 0.05).

### 3.4 Integrated CMJ responses assessed by area under the curve following individualized drop-jump conditioning

Although not statistically significant, the caffeine condition exhibited numerically higher total AUC and positive AUC values compared with placebo, whereas negative AUC values were comparable between conditions. The confidence intervals for all AUC comparisons included zero, indicating no clear evidence of a condition-related difference in cumulative CMJ responses during the post-conditioning period.

Exploratory analysis of individual net benefit responses (CAF − PLA) demonstrated heterogeneous response patterns among participants. Individual differences varied across participants and assessment time points, with some athletes displaying positive caffeine-related changes, whereas others showed minimal differences or negative changes relative to placebo (**Figure 5**, **Table 5**).

**Figure 5:**
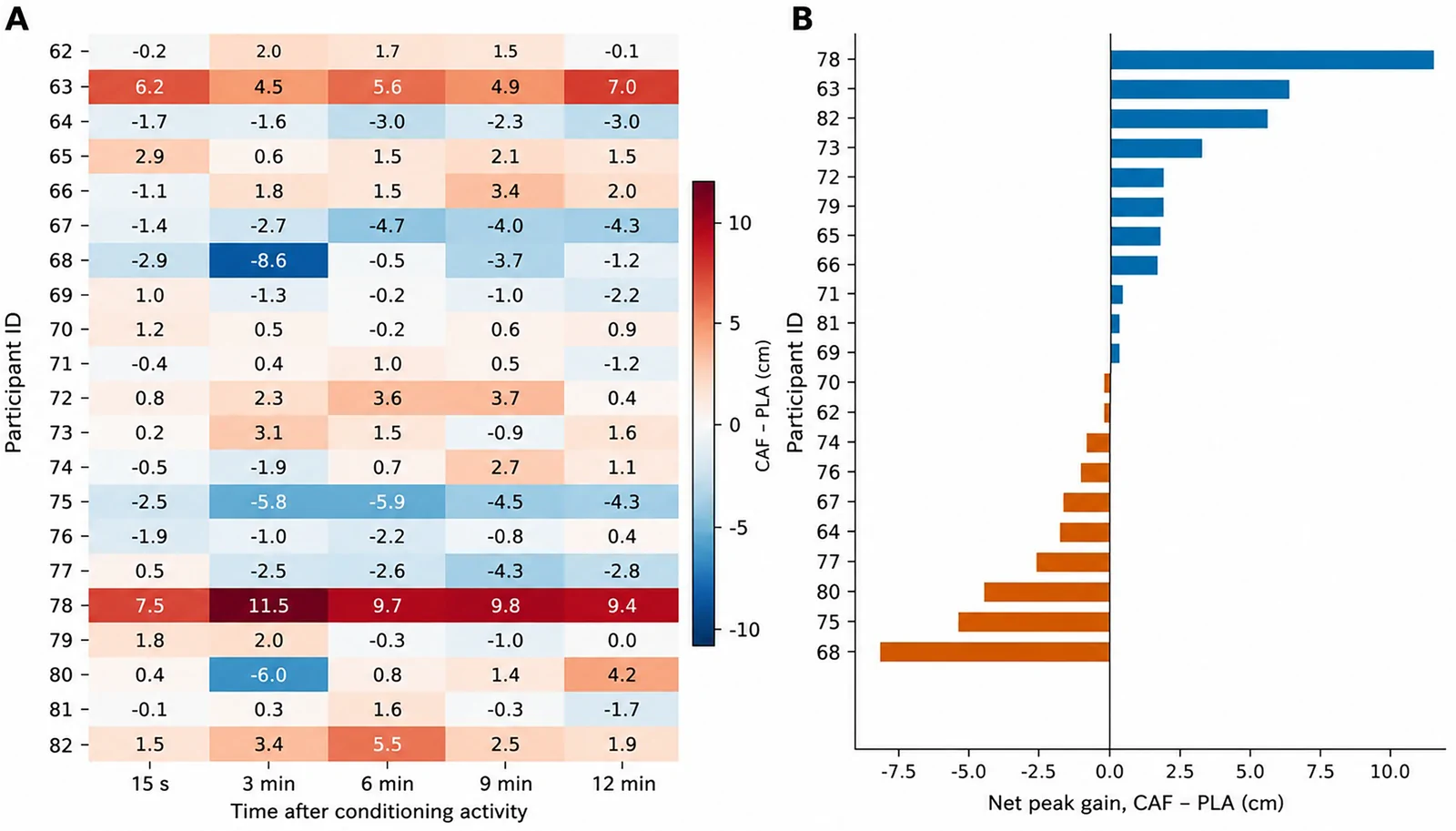
Individual variability in caffeine-related net CMJ performance responses following individualized DJ conditioning. **(A)** Heatmap illustrating the individual net CMJ performance gain (CAF − PLA, cm) at each post-conditioning assessment time point (15 s, 3 min, 6 min, 9 min, and 12 min). Positive values (red) indicate greater CMJ performance following caffeine ingestion compared with placebo, whereas negative values (blue) indicate lower performance under caffeine. Participants are displayed individually according to participant ID. **(B)** Horizontal bar plot showing each participant’s net peak CMJ performance gain (CAF − PLA), calculated as the difference between the maximal CMJ improvement achieved under caffeine and placebo across all post-conditioning time points. Participants are ranked from the largest positive to the largest negative net gain.

**Table 5:** Cumulative CMJ response profiles quantified by AUC following individualized DJ conditioning under caffeine and placebo conditions.

Outcome	Caffeine	Placebo	Mean difference	95% CI	t(df)	p
Total AUC (cm·min)	8.12	2.93	5.19	−11.27 to 21.66	0.62 (20)	0.544
Positive AUC (cm·min)	16.58	11.65	4.93	−9.35 to 19.21	0.68 (20)	0.507
Negative AUC (cm·min)	−8.46	−8.72	0.26	−5.11 to 5.64	0.10 (20)	0.925

Data are presented as mean ± standard error (SE). Between-condition comparisons were performed using paired-samples t-tests. The area under the curve (AUC) was calculated using the trapezoidal method based on CMJ changes relative to the post-warm-up baseline. Total AUC represents the overall cumulative CMJ response, whereas positive AUC and negative AUC represent the accumulated periods of performance enhancement and decrement relative to baseline, respectively. As total AUC is derived from the sum of positive and negative AUC components, these variables were interpreted as related measures within the same comparison framework. Statistical significance was set at an adjusted p-value < 0.05.

Collectively, AUC analyses indicated that acute caffeine ingestion did not significantly alter the cumulative magnitude of CMJ performance responses following individualized drop-jump conditioning. However, individual response profiles revealed variability in the magnitude and direction of caffeine-related changes across athletes.

### 3.5 Sensitivity analyses of temporal CMJ responses using linear mixed-effects models and leave-one-participant-out analysis

As a complementary sensitivity analysis to the primary repeated-measures ANOVA, linear mixed-effects models (LMMs) were used to determine whether the temporal pattern of CMJ responses was sensitive to the covariance structure used to account for within-participant serial correlation. For absolute CMJ, the condition × time interaction was not significant with either an AR(1) covariance structure (χ²(6) = 3.072, p = 0.800) or an unstructured (UN) covariance structure (p = 0.681). Similarly, for ΔCMJ, the condition × time interaction remained non-significant under both AR(1) (χ²(4) = 2.409, p = 0.661) and UN (p = 0.697) covariance structures. These findings were consistent with the primary repeated-measures ANOVA, indicating that the absence of a differential temporal response between CAF and PLA was not dependent on the specified covariance structure (**Table 6**).

**Table 6:** Sensitivity analysis of the condition × time interaction for temporal CMJ responses using alternative covariance structures.

Outcome	Covariance structure	Condition × time interaction, p
Absolute CMJ	AR(1)	0.800
Absolute CMJ	UN	0.681
ΔCMJ	AR(1)	0.661
ΔCMJ	UN	0.697

AR(1), first-order autoregressive covariance structure; UN, unstructured covariance structure; CMJ, countermovement jump; ΔCMJ, change in CMJ height relative to the post-warm-up baseline. p values represent two-sided tests of the condition × time interaction. The LMMs included condition, time, and condition × time as fixed effects and participant as a random intercept. The table presents the covariance-structure sensitivity analysis; detailed model specifications are provided in the Data Analysis section.

To further evaluate whether the temporal response pattern was disproportionately influenced by any individual participant, LOPO sensitivity analysis was conducted within the same LMM framework. Each participant was sequentially excluded, and the model was refitted using the remaining 20 participants. Across all 21 LOPO iterations, the overall CMJ response pattern following individualized drop-jump conditioning remained stable. Likelihood ratio tests for the condition × time interaction were consistently non-significant following the removal of any single participant (χ² range = 0.672–1.757, df = 5, all p > 0.88), indicating that no individual participant disproportionately influenced the temporal response pattern.

The estimated caffeine-related differences in CMJ performance also remained directionally consistent across LOPO iterations. The estimated effects at 15 s, 6 min, 9 min, and 12 min were consistently positive, with coefficient ranges of 0.19–0.71 cm, 0.27–1.05 cm, 0.03–0.74 cm, and 0.01–0.70 cm, respectively. Although the estimated effect at 3 min showed greater variability (−0.53 to 0.48 cm), exclusion of any single participant did not alter the overall interpretation of the findings (**Figure 6**).

**Figure 6:**
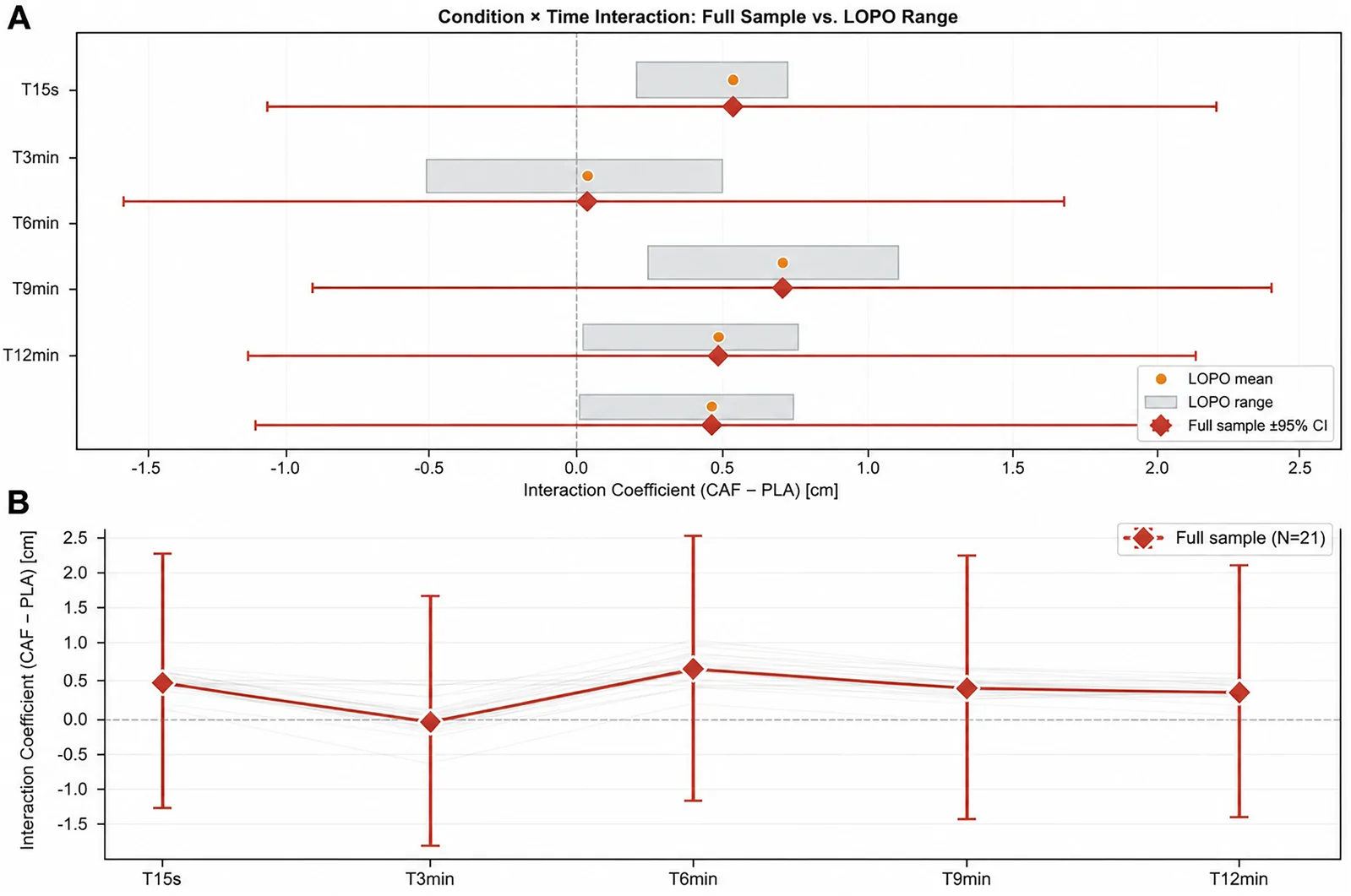
Leave-one-participant-out sensitivity analysis of the temporal CMJ response following individualized DJ conditioning. **(A)** Comparison of the estimated condition-by-time interaction coefficients (CAF − PLA) obtained from the full-sample mixed-effects model and the leave-one-participant-out (LOPO) analyses. Red diamonds and horizontal error bars represent the full-sample estimates with 95% confidence intervals, whereas orange circles and shaded rectangles indicate the mean and range of the corresponding estimates across all LOPO iterations, respectively. The vertical dashed line denotes the null effect (0 cm). **(B)** Estimated condition-by-time interaction coefficients across all LOPO iterations. The red line and diamonds represent the full-sample estimates, whereas the gray lines indicate the corresponding estimates obtained after sequential exclusion of each individual participant.

Taken together, the LMM and LOPO sensitivity analyses supported the robustness of the temporal CMJ response pattern, indicating that the absence of a significant condition × time interaction was neither dependent on the assumed covariance structure nor disproportionately driven by any individual participant.

## 4 Discussion

The present randomized, double-blind, placebo-controlled crossover study investigated whether acute caffeine ingestion further modifies the temporal and multidimensional characteristics of PAPE following RSI-guided individualized DJ conditioning in high-level male basketball players. The principal findings were that caffeine ingestion at 3 mg·kg^-1^ did not significantly modify the temporal profile of CMJ performance or the multidimensional characteristics of PAPE compared with placebo. Although CMJ performance showed numerically higher values under caffeine at several recovery time points, no significant between-condition differences were observed in peak performance, time to peak performance, cumulative response, or response robustness. Considerable inter-individual variability was observed in the magnitude and timing of performance responses, whereas leave-one-participant-out sensitivity analysis supported the stability of the observed temporal pattern. Collectively, these findings suggest that, under the conditions examined, acute caffeine ingestion at 3 mg·kg^-1^ did not produce a detectable additional enhancement of PAPE following RSI-guided individualized DJ conditioning at the group level. These findings highlight that the contribution of caffeine to PAPE may depend on factors such as dose, conditioning stimulus characteristics, and individual athlete responsiveness.

The transient temporal pattern observed following individualized DJ conditioning is consistent with the dynamic nature of PAPE, in which performance reflects the time-dependent interaction between potentiation and residual fatigue rather than a uniform enhancement across recovery intervals. The magnitude and temporal characteristics of PAPE may be influenced by several factors, including training status, reactive strength, conditioning intensity, recovery duration, and individual neuromuscular capacity (Blazevich and Babault, 2019; Seitz and Haff, 2016; Wilson et al., 2013; Xu et al., 2025). In this context, prescribing DJ height according to each athlete’s RSI may help align the conditioning stimulus with individual reactive strength characteristics while limiting unnecessary mechanical loading. Such stimulus individualization may facilitate an appropriate potentiation–fatigue balance and could partly explain the transient performance profile observed in the present study. However, because the present study did not include a non-individualized DJ condition, the extent to which RSI-guided individualization itself optimized PAPE cannot be determined directly.

Despite the well-established ergogenic effects of caffeine on exercise performance, acute caffeine ingestion at 3 mg·kg^-1^ did not further enhance CMJ performance following RSI-guided individualized DJ conditioning in the present study. This finding is consistent with previous evidence showing that caffeine does not necessarily augment lower-body PAPE when an effective conditioning stimulus has already been applied, particularly in well-trained athletes (Davis and Green, 2009; Filip-Stachnik et al., 2022; Guest et al., 2021; Lazić et al., 2022). One possible explanation is that RSI-guided individualization may have provided a conditioning stimulus closely matched to each athlete’s reactive strength capacity, thereby limiting unnecessary mechanical loading and reducing the scope for further acute enhancement. This interpretation is also compatible with the relatively high neuromuscular capacity expected in Tier 3 (Highly Trained/National Level) athletes, for whom an appropriately prescribed DJ stimulus may already elicit substantial neuromuscular activation. However, these mechanisms should be interpreted cautiously because the present study did not include a non-individualized DJ condition or direct measures of neuromuscular activation. Thus, the absence of a significant caffeine effect should not be interpreted as evidence that caffeine is ineffective, but rather that, at 3 mg·kg^-1^ and under the specific conditioning and participant characteristics examined here, its additional ergogenic contribution may have been limited.

A further explanation may relate to the partial mechanistic overlap between caffeine-induced ergogenic effects and the neuromuscular processes underlying PAPE. Caffeine primarily acts through adenosine receptor antagonism, which may increase neural drive, motor unit recruitment, and corticospinal excitability, whereas explosive conditioning activities can enhance performance through increased neural activation, musculotendinous stiffness, muscle contractile function, and stretch-shortening cycle efficiency (Boullosa, 2021; Davis and Green, 2009; Guest et al., 2021; Tallis et al., 2015). When an appropriately individualized DJ stimulus has already elicited substantial neuromuscular activation, the additional contribution of caffeine may therefore be relatively small and difficult to detect at the group level. This interpretation may also help explain the substantial inter-individual variability observed in the present study. Factors such as habitual caffeine exposure, genetic variability, and caffeine sensitivity may contribute to differences in individual responsiveness (Guest et al., 2021; Jump et al., 2026; Pickering and Kiely, 2018, 2019). Recent evidence has further demonstrated heterogeneous vertical jump responses among athletes following caffeine ingestion, suggesting that even standardized relative dosing may not result in uniform ergogenic effects across individuals (Papadakis et al., 2025). Testing time may also influence the magnitude of caffeine-related performance effects (Mora-Rodríguez et al., 2015). Because habitual caffeine intake, caffeine-related genetic characteristics, and circadian responses were not directly assessed in the present study, these factors should be regarded as potential contributors to response heterogeneity rather than established explanations for the absence of a group-level ergogenic effect.

Beyond group-level comparisons, the multidimensional analyses provided additional insight into the temporal and individual characteristics of PAPE. Although no significant between-condition differences were observed in peak response magnitude or time to peak, peak CMJ performance occurred numerically earlier under caffeine, with nearly half of the participants reaching their individual peak at 15 s, whereas peak responses under placebo were more frequently observed at 3 min. This distribution suggests that the timing of maximal performance enhancement may vary substantially among athletes and may be influenced by the interaction between caffeine ingestion and the conditioning stimulus, although the present data do not establish a statistically significant acceleration of the PAPE response (Guest et al., 2021; Seitz and Haff, 2016; Tallis et al., 2015; Wilson et al., 2013; Xu et al., 2025). AUC analysis further complemented the time-point comparisons by quantifying the integrated CMJ response across the 12-min recovery period. Although caffeine did not significantly increase total, positive, or negative AUC, the numerical differences in favor of caffeine indicate that any potential cumulative advantage was small and insufficient to produce a detectable group-level effect. Importantly, the LOPO sensitivity analysis showed that sequential exclusion of individual participants had minimal influence on the observed temporal estimates, supporting the robustness of the overall response pattern and indicating that the principal findings were not driven by a single participant.

From a practical perspective, the present findings support an individualized rather than additive approach to PAPE preparation in high-level basketball. For practitioners, RSI-guided DJ conditioning may provide a practical method for selecting an athlete-specific drop height and matching the conditioning stimulus to neuromuscular readiness before explosive activities such as jumping, sprinting, or competition. Given the variability in peak magnitude and timing observed among athletes, recovery duration should also be individualized and determined through repeated monitoring of CMJ performance rather than relying on a fixed post-conditioning interval (Cengizel, 2025; Wilson et al., 2013; Xu et al., 2025). Importantly, the absence of a significant additional effect at 3 mg·kg^-1^ suggests that caffeine should not be assumed to enhance an already individualized PAPE protocol in high-level male basketball players. Instead, caffeine may be considered as an optional ergogenic strategy, with its use guided by the athlete’s individual response, habitual practices, and the specific demands of the performance setting (Guest et al., 2021; Pickering and Kiely, 2018).

Several limitations should be considered when interpreting these findings. First, the relatively small sample size may have limited the sensitivity to detect small condition × time effects and restricts the generalizability of the findings beyond Tier 3 male basketball players. The *a priori* sample-size calculation assumed a medium effect (f = 0.25) and ϵ = 1.0, whereas the observed condition × time interaction was smaller (ηp² ≈ 0.02–0.03; f ≈ 0.15–0.16) and the sphericity assumption was violated. Although Greenhouse–Geisser correction was applied where appropriate, the present sample may have had limited sensitivity to detect small caffeine-related differences. Thus, the nonsignificant interaction should not be interpreted as definitive evidence that caffeine cannot produce a small additional effect on PAPE. Larger, adequately powered studies involving female athletes, athletes of different performance levels, and other sports are warranted to determine the generalizability and reproducibility of these findings. Second, habitual caffeine intake, caffeine sensitivity, and caffeine-related genetic characteristics, such as CYP1A2 polymorphisms, were not assessed. These factors may contribute to inter-individual variability in caffeine responsiveness and limit the ability to explain the heterogeneous individual responses observed. Third, repeated maximal CMJ assessments within the same experimental session may have influenced subsequent responses, as later time points were preceded by multiple maximal jumping efforts. Although the testing procedure was standardized across participants, time points, and experimental conditions, the observed temporal trajectory may therefore partly reflect the cumulative effects of repeated CMJ testing. Future studies could assess individual post-conditioning recovery time points in separate randomized sessions to better isolate responses at each interval, while recognizing the potential for greater between-day variability. Fourth, the study relied primarily on CMJ performance and did not include direct measures of neuromuscular function, such as electromyography or corticospinal excitability. Consequently, the physiological mechanisms underlying the interaction between acute caffeine ingestion and RSI-guided individualized DJ conditioning cannot be established directly. Future research should integrate individualized conditioning protocols with larger samples, characterization of caffeine-related individual factors, and direct neuromuscular assessments to clarify which athletes may benefit from combined caffeine and PAPE strategies and under which conditions.

## 5 Conclusion

The present randomized, double-blind, placebo-controlled crossover study investigated whether acute caffeine ingestion further modifies PAPE responses following RSI-guided individualized DJ conditioning in high-level male basketball players. At a dose of 3 mg·kg^-1^ body mass, acute caffeine ingestion did not produce a detectable additional enhancement of CMJ performance compared with placebo under the conditions examined. Although caffeine resulted in numerically higher CMJ values at several recovery time points, no significant between-condition differences were observed in the temporal profile, peak response magnitude, cumulative response, or robustness of the PAPE response. These findings suggest that, within the context of the present protocol and participant characteristics, caffeine at the investigated dose may provide limited additional benefit when combined with RSI-guided individualized DJ conditioning. Future research should investigate whether different caffeine doses, habitual caffeine exposure, caffeine metabolism-related genetic factors, and neuromuscular characteristics contribute to variability in individual responses to combined caffeine and PAPE interventions.

## Data Availability

The raw data supporting the conclusions of this article will be made available by the authors, without undue reservation.
